# A 5-Month-Old Infant with Right Scrotum Swelling; a Case Report

**Published:** 2017-01-14

**Authors:** Shih-Wen Hung, Kuo-Chih Chen, Chin-Chu Wu, Tzong-Luen Wang, Aming Chor-Ming Lin

**Affiliations:** 1 Emergency Department, Shin Kong Wu Ho-Su Memorial Hospital, Taipei, Taiwan.; 2 Department of Medical Imaging, Shin Kong Wu Ho-Su Memorial Hospital, Taipei, Taiwan.; 3 School of Medicine, Fu-Jen Catholic University, New Taipei City, Taiwan.

**Keywords:** Ventriculoperitoneal shunt, equipment failure, hydrocephalus, radiography, abdominal, case reports

## Abstract

Migration of the distal catheters of ventriculoperitoneal (VP) shunt is a rare event. Here, we report an unusual case of a 5-month-old infant with post-hemorrhagic hydrocephalus, who developed right scrotum swelling soon after VP shunting. Plain abdominal x-ray showed the shunt tubing, which was twisted and kinked in its distal portion and coiled in the right scrotum. The infant was operated on and managed with successful outcome.

## Introduction

Ventriculoperitoneal (VP) shunting is one of the most common pediatric neurosurgery operations performed for dealing with hydrocephalus (1). Following the performance of this procedure, excess cerebrospinal fluid (CSF) will be diverted from the brain ventricles to the peritoneal cavity of the abdomen via a catheter (2). However, it can be associated with numerous complications and consequences such as infection, knotting, malfunction and etc. that are reported to have a rate as high as 70% in the first year after placement and an annual occurrence rate of about 5% thereafter (2, 3). Migration of the distal tube of VP shunt into several body compartments are among rare complications of VP shunting, which have been reported as case reports in existing literature (4-7). Here we report a case of distal portion migration of VP shunt through the processus vaginalis into the scrotum. 

## Case presentation:

**Figure 1:**
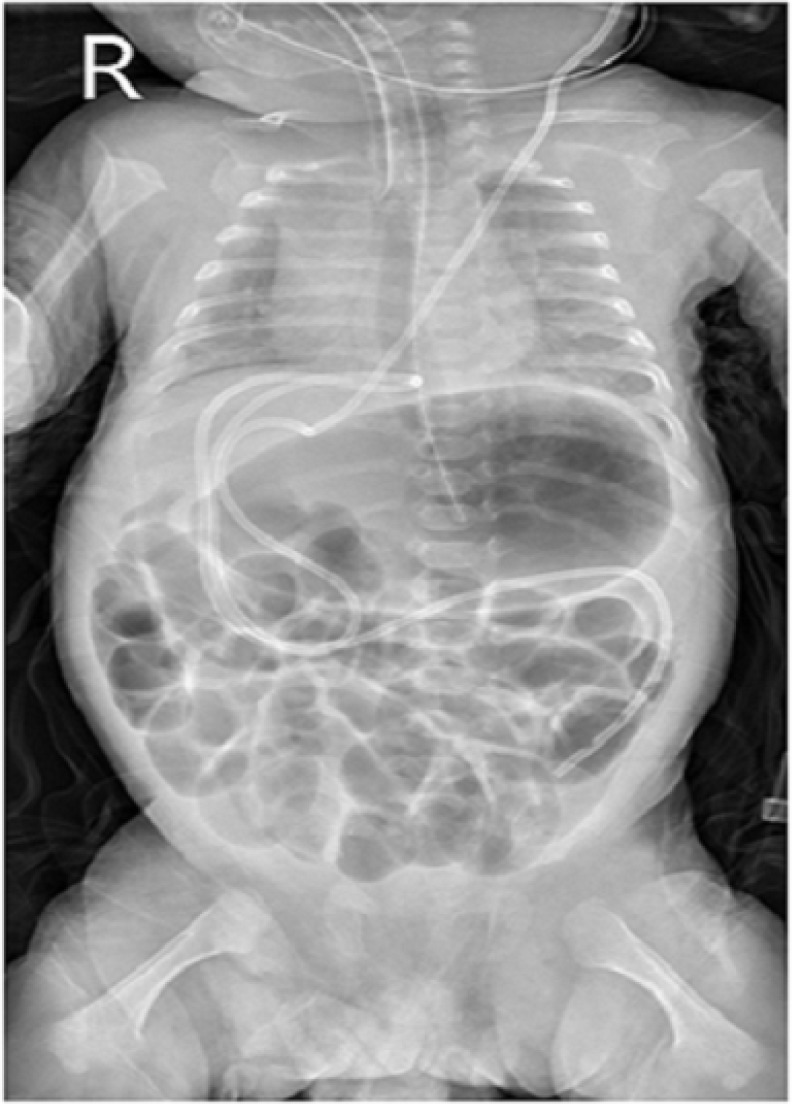
Abdominal radiography of patients after ventriculo-peritoneal shunt placement

A five-month-old male infant was referred to the emergency department with history of right scrotal swelling, which had slowly grown over several days. There was no history of vomiting, diarrhea, hematuria, irritability or scrotum trauma. The patient was born at 28 weeks gestational age, with birth-weight of 1020 gr. As a result of prematurity, he had post-hemorrhagic hydrocephalus and a left sided VP shunt was placed 3 months after birth. Check-up frontal radiography of the chest and abdomen performed after operation is shown in figure 1. 

On arrival, vital signs were as follows: temperature 36°c, pulse rate 120 beats/minute, respiratory rate 30 breaths/min, blood pressure 100/62 mmHg and SaO_2_ 99%. In physical examination, he appeared malnourished. The right scrotum was found to be distended. Bilateral testicles were palpable on both sides. There were no features of shunt malfunction. 

A complete blood cell count showed the following: leukocyte count 7900/mm3; segmented neutrophils 65%; hemoglobin level 9.3 mg/dL; hematocrit 25.9%; and platelet 190000/uL. Other laboratory studies included: glucose 92 mg/dL; serum urea nitrogen 10 mg/dL; serum creatinine 0.2 mg/dL; sodium 140 mEq/L; potassium 3.9 mEq/L; C-reactive protein 2.9mg/L; and prothrombin time with an international normalized ratio of 1.2. 

**Figure 2:**
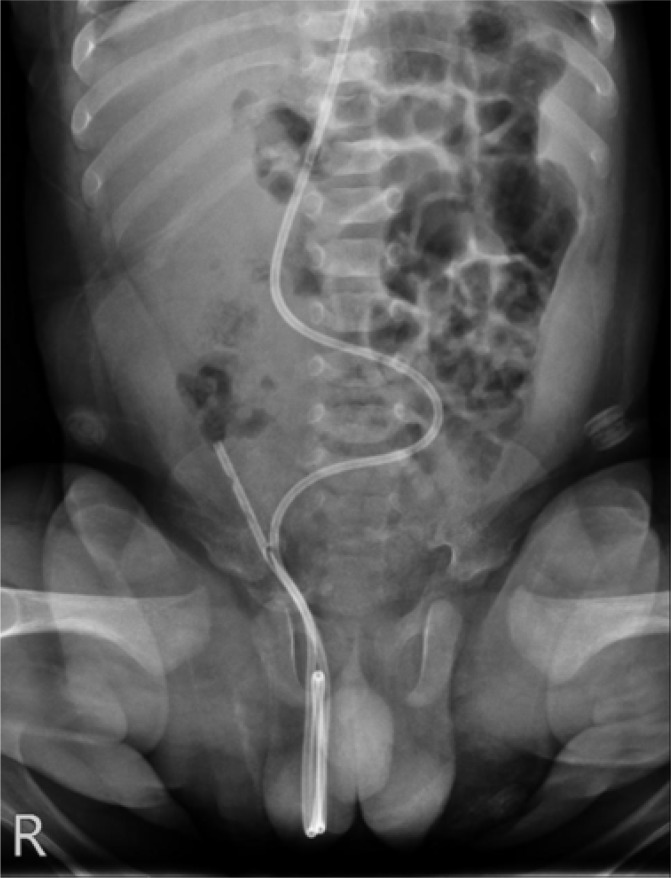
Abdominal radiography of patient in emergency department

The patient underwent an abdomen x-ray that is shown in figure 2. Abdomen x-ray showed the shunt tube in the abdomen, which was twisted and kinked in its distal portion and coiled in the right scrotum. 

Operation was performed by right groin incision. The peritoneal end of the shunt was repositioned in the peritoneal cavity, and the processus vaginalis was closed. The patient had an uneventful postoperative recovery and was discharged four days later.

## Discussion

VP shunts are catheters that are inserted into the ventricles within the brain and threaded under the skin from the skull to the peritoneum, where excess CSF is drained (2). VP shunting for hydrocephalus is one of the most common operations in pediatric neurosurgery. Shunt is placed to divert CSF from the dilated ventricles to the peritoneal cavity, where it is absorbed. 

VP shunting is associated with well-known complications including over drainage, obstruction or malfunction, infection, coiling, migration, knotting and viscous perforation (8-11). VP shunt complications are classified as mechanical, functional and infective. Shunt infection is the most common post-operative complication. Another common complication of a VP shunt is shunt obstruction or malfunction. The definitive treatment for both infection and shunt obstruction is surgical shunt revision. A proper clinical assessment leads to timely identification of complications and their prompt treatment. Infants with VP shunts should be monitored lifelong by neurosurgeons.

Migration of the distal catheters of VP shunt is a rare complication. The distal catheter of VP shunt can migrate into various body parts. Migration of VP shunt has been reported in several body compartments, including the mediastinum, chest, abdominal wall, gastrointestinal tract, and pelvic cavity (11, 12). Common presentations of VP shunt malposition complications include peritonitis, gastrointestinal perforation, ileus, inguinal hernia, peritoneal pseudocysts, loss of catheter into the peritoneal cavity, and abscesses. Most of these patients present with abdominal signs. Migration occurs when the shunt tube moves from its original position to a location that inhibits proper drainage. Migration of the distal portion of the shunt through the processus vaginalis into the scrotum is a very rare complication (13). The migration of a tube into the scrotum also requires a patent processus vaginalis. This event can be subsequent to high intra-abdominal pressure causing migration of the catheter into scrotum. The length of the distal catheter in peritoneal cavity raises the probability of migration. Migration can occur due to improper length. In our case, the plain abdominal x-ray showed the shunt tube, which was twisted and kinked in its distal portion and coiled in the right scrotum. Gupta M et al. investigated 30 children with full-length peritoneal shunts to recognize the rate of complications. Out of the 30 children, the minimum length of the distal catheter placed in peritoneal cavity was 44 cm and the maximum length was 52 cm. It was shown that use of an extended length peritoneal shunt catheter was not associated with an increase in complications and eliminated the need to lengthen the peritoneal catheter for growth of the patient (14). Long-term outcome of VP shunt placement in infants revealed a relatively high rate of complications requiring shunt revision as late as 30 years after initial placement (15).

Usually the primary image is easily accessible and frequently compared to newly taken images to diagnose migration of the distal catheter of VP shunt. 

A high index of suspicion is required when making the diagnosis. Diagnosis can be established by plain x-ray of the abdomen, which identifies the distal portion of the shunt tube in the scrotum. Prompt surgical management for catheter repositioning is recommended in these cases to avoid the risk of further complications.
